# Personality in women and associations with mortality: a 40-year follow-up in the Population Study of Women in Gothenburg

**DOI:** 10.1186/1472-6874-14-61

**Published:** 2014-04-30

**Authors:** Malin André, Eva Billstedt, Calle Bengtsson, Tore Hällström, Lauren Lissner, Ingmar Skoog, Valter Sundh, Margda Waern, Cecilia Björkelund

**Affiliations:** 1 Department of Primary Health Care, Institute of Medicine, Sahlgrenska Academy, University of Gothenburg, Box 454, S-405 30 Gothenburg, Sweden; 2 Department of Medical and Health Sciences, Linköping University, Linkoping, Sweden; 3 Public Health Epidemiology, Department of Public Health and Community Medicine, Sahlgrenska Academy, University of Gothenburg, Gothenburg, Sweden; 4 Department of Neuroscience and Physiology, Section of Psychiatry and Neurochemistry, Unit for Neuropsychiatric Epidemiology, Sahlgrenska Academy, University of Gothenburg, Gothenburg, Sweden

**Keywords:** Personality traits, Secular trends, Population-based cohort, Women, Longevity

## Abstract

**Background:**

The question of whether personality traits influence health has long been a focus for research and discussion. Therefore, this study was undertaken to examine possible associations between personality traits and mortality in women.

**Methods:**

A population-based sample of women aged 38, 46, 50 and 54 years at initial examination in 1968–69 was followed over the course of 40 years. At baseline, 589 women completed the Cesarec-Marke Personality Schedule (the Swedish version of the Edwards Personal Preference Schedule) and the Eysenck Personality Inventory. Associations between personality traits and mortality were tested using Cox proportional hazards models.

**Results:**

No linear associations between personality traits or factor indices and mortality were found. When comparing the lowest (Q1) and highest quartile (Q4) against the two middle quartiles (Q2 + Q3), the personality trait Succorance Q1 versus Q2 + Q3 showed hazard ratio (HR) = 1.37 (confidence interval (CI) = 1.08-1.74), and for the factor index Aggressive non-conformance, both the lowest and highest quartiles had a significantly higher risk of death compared to Q2 + Q3: for Q1 HR = 1.32 (CI = 1.03-1.68) and for Q4 HR = 1.36 (CI = 1.06-1.77). Neither Neuroticism nor Extraversion predicted total mortality.

**Conclusions:**

Personality traits did not influence long term mortality in this population sample of women followed for 40 years from mid- to late life. One explanation may be that personality in women becomes more circumscribed due to the social constraints generated by the role of women in society.

## Background

The question of whether personality traits influence health has long been a focus of research and discussion. Since the studies in the 1970s by Friedman and Rosenman claiming that persons with a type A personality are likely to suffer from coronary heart disease, several prospective population-based studies have been carried out [1,2]. Later studies have not been able to confirm these findings. On the contrary, one study surprisingly found a protective effect of type A behaviour for women [3]. Besides type A personality, hostility and neuroticism have been associated with higher mortality in longitudinal studies. Hostility and chronic anger were described as risk factors for cardiovascular disease and premature mortality [4,5]. However, results of studies concerning neuroticism as a predictor for mortality were inconsistent [6-11]. No association between personality and cancer has been convincingly demonstrated [12].

In recent years, the Five-Factor Model of personality has been recognised as a reference for studies of personality and health outcomes [13]. Instead of focussing on risk factors for mortality, factors associated with longevity, above all conscientiousness, have been identified. However, there are still no published population-based studies in this field with a time span of more than a decade [11,14].

The mechanisms linking personality to health outcomes are discussed in different models, as reported in the recent review by Chapman et al. [2]. However, the importance of gender aspects has not always been considered. Personality traits may be associated with the factors that cause disease – for example, cardiovascular reactivity and immune responses – or personality traits may influence health behaviour. Moreover, personality traits may influence how individuals cope with life stress and adhere to treatment regimes [15-17].

In the Population Study of Women in Gothenburg 1968–69 [18], personality traits were studied with the Eysenck Personality Inventory (EPI) and the Cesarec-Marke Personality Schedule (CMPS). The EPI measures the two personality dimensions extra/introversion and neuroticism [19]. A high score on the EPI Neuroticism scale indicates emotional reactivity, low ego strength, guilt proneness and anxiety, whereas high scores on the Extraversion scale indicate sociable, outgoing, impulsive and uninhibited behaviour. The Lie scale included in the EPI reflects a tendency to present oneself in a socially desirable manner. The CMPS is based on Murray’s theory of personality [20]. Murray defined two kinds of needs: primary, such as hunger, thirst and sexuality, and secondary, such as wishes and pursuits. From these secondary needs an inventory was developed – the Edward’s Personal Preference Schedule (EPPS). The CMPS is a Swedish version of the EPPS [21-23].

A cross-sectional study from 1969 showed that the level of aggression and neurotic self-assertiveness was higher in women with myocardial infarction than in the general population [21]. In a 12-year follow-up of the Population Study of Women in Gothenburg, no association between personality traits and death rate was reported, but low ratings of guilt feelings and neurotic self-assertiveness were predictive of myocardial infarction [22]. A longer follow-up time – at present, 4 decades since baseline – provides the possibility for further analyses of personality traits and mortality.

The aim of this study was to examine possible associations between personality traits and mortality over a 40-year period in a representative sample of Swedish urban women aged 38, 46, 50 and 54 when first examined in 1968–69.

## Methods

### Study population

#### Participants in the Population Study of Women in Gothenburg

As part of the Population Study of Women in Gothenburg in 1968–69, a representative sample of 800 middle-aged women living in Gothenburg, Sweden, were invited to have a free health examination and a comprehensive psychiatric examination [18]. The sample was obtained from the Internal Revenue Office Register and the sampling method was based on birthdays to make it representative. The cohorts were aged 38, 46, 50 and 54. Among the 38-year-old women, those born on day 12 of each month and day 18 of each odd month participated; among 46- and 50-year-olds, all women born on days 12, 18, 24 and 30 of each month participated; and among 54-year-olds, all women born on day 12 of each month participated [24]. The CMPS and EPI (self report) were not introduced until the investigation had been underway for 2.5 months, which resulted in a data absence of 22% for women born in the early months of the year. Women who were not able to fill out the personality inventories due to language problems, severe intelligence defects or mental illness were not included in the examination. All together, 589 women 38–54 years of age (74%) filled out the CMPS and EPI personality instruments.

#### Social variables

Education was classified as elementary (corresponding to 6 years in those born from 1914 to 1922 and 7 years in those born in 1930), high school (corresponding to a total of at least 7 to 9 years), or >9 years + academic (university). Women reported their own occupation and income and, if they were married, their husbands’ occupation and income. This information was transformed according to a standard occupation grouping system into the following social group classification: socioeconomic group 1 (large-scale employers and officials of high or intermediate rank), group 2 (small-scale employers, lower-rank officials and supervisors), and group 3 (skilled and unskilled workers). Information concerning marital status (single, married, divorced or widowed) was obtained from the registrar’s office in 1968–69. The women reported the number of their children living at home. Subjects who had never smoked or stopped smoking more than a year before the examination in 1968–69 were classified as non-smokers. Concerning alcohol, subjects were classified as consumers if they had consumed beer, wine or spirits some days per month or more during the previous year. Subjects were classified as being physically inactive during leisure time if they reported usually spending less than 4hours a week gardening, running, dancing, playing golf or tennis or engaged in similar activities during the previous year.

#### Data on mortality

Information on mortality from 1968–2008 was obtained from the National Swedish Death Registry.

#### Data access

The data is owned by the Faculty of Sahlgrenska Academy, University of Gothenburg and according to the Swedish regulations not publically available.

### Personality investigation

The CMPS is a questionnaire with 165 questions answered with the alternatives “Yes” or “No”. It has 11 subscales each consisting of 15 questions. The subscales and what they measure are:

1. Achievement (ACH): need to accomplish something important and difficult, and need to compete with and surpass others.

2. Affiliation (AFF): need to form close emotional relations, and to adhere and remain loyal to friends.

3. Aggression (AGG): need to revenge an injury, and impulsive aggression and irritability.

4. Defence of status (DST): need to defend one’s status, sensitivity to the opinions of others, and tendency to refrain from actions in order to avoid failure.

5. Guilt feelings (GUI): guilt feelings, and a strict conscience with a strong sense of duty.

6. Dominance (DOM): need to dominate and lead others.

7. Exhibition (EXH): need to expose oneself, to be in the centre and be noticed.

8. Autonomy (AUT): need to be independent, to disregard the opinions of others and avoid responsibility.

9. Nurturance (NUR): need to help, nurse and take care of others.

10. Order (ORD): need for order, cleanliness and planning.

11. Succorance (SUC): need to be taken care of and be helped both emotionally and practically.

Five factor indices, based on factor analyses, are calculated. They are considered a summary on a more general level [20]:

i. Neurotic self-assertiveness

ii. Dominance

iii. Aggressive non-conformance

iv. Passive dependency

v. Sociability

The EPI measures the personality dimensions extra/introversion and neuroticism with 24 questions answered by “Yes” or “No” [19]. The inventory also includes a “Lie scale” of nine questions.

Scores from 1968–69 were calculated from the data according to an algorithm [20].

### Statistical methods

The associations between personality factors and mortality were analysed both as linear associations (by quartile of personality trait) and by comparing the lowest and highest quartiles separately with the two middle quartiles using Cox proportional hazard models Estimation of Hazard Ratio was made in risk time model from Poisson regression with event variable “Dead within 40 years” and risk time variable “Survival days 1968 + 40 years”. Results are reported as a hazard ratio (HR) and a 95% confidence interval (CI). Age at baseline was used as a covariate in all models.

The interaction between personality by time and mortality (i.e., non-proportional hazard) was tested by adding an interaction term (time*personality) to the model and using the change in -2 log likelihood of the model as a test statistic. No significant interaction with time was found in any analysis. We also calculated separate models for different time periods to examine the stability of the associations. The same procedure was used for examining the interaction between personality by age and mortality. Difference between younger and older women concerning personality traits was tested with a *t*-test.

The Ethics Committee of the University of Gothenburg approved the study.

## Results

Table 1 describes participating women in the different age groups. There were no statistically significant differences concerning marital status, education, socioeconomic status, leisure time activity or smoking between the subgroups selected or not selected for the psychiatric examination [18].

**Table 1:** **Participants in the 1968–69 examination for the Population Study of Women in Gothenburg; relevant descriptive data in 1968**–**69 concerning cohorts taking part in the Cesarec**-**Marke Personality Schedule and Eysenck Personality Inventory instrument examination**

	**Age 38 n = 87 (%)**	**Age 46 n = 219 (%)**	**Age 50 n = 215 (%)**	**Age 54 n = 68 (%)**	**Total n = 589 (%)**
Mortality					
Dead within 40 years (n (%))	24(27.6)	123(56.2)	156(72.6)	59(86.8)	362(61.5)
Marital status					
Unmarried	5.7	6.9	6.5	4.4	6.3
Married	85.0	82.9	80.9	75.0	81.6
Divorced	8.0	7.8	6.0	10.3	7.5
Widowed	1.1	2.3	6.5	10.3	4.6
Socioeconomic group					
1	14.1	17.8	11.0	13.1	14.3
2	50.6	44.1	43.1	39.3	44.2
3	35.3	38.0	45.9	47.5	41.5
No children at home (%)	13.8	13.8	17.7	13.2	15.2
Education					
Elementary school	66.7	68.1	73.0	77.6	70.8
High school	25.3	24.5	23.0	22.4	24.1
Academic education	8.0	7.4	3.3	0	
Alcohol (% yes)					
Beer (≥some days per month)	72.4	68.2	64.7	61.9	66.8
Wine (≥some days per month)	52.9	50.7	54.0	52.5	52.3
Liquor (≥some days per month)	24.1	24.0	30.7	27.9	26.9
Physically inactive during leisure time (% yes)	14.9	17.1	20.9	17.6	18.2
Smoking (% yes)	44.8	40.6	37.7	45.6	40.7

Table 2 describes differences between the age groups for personality traits in the 1968–69 examination. There was a significant trend for lower scores among younger age groups concerning Affiliation, Nurturance, Sociability and the Lie scale, while the trend concerning Aggression was the opposite. In all other personality traits there were no significant differences between age groups.

**Table 2:** **Mean values and standard deviations for personality traits and factor indices for women participating in the psychiatric examination of the 1968**–**69 Population Study of Women in Gothenburg as well as differences in means between younger** (**aged 38**–**46**) **and older** (**aged 50**–**54**) **age groups with a 95% confidence interval (CI) for age group difference**

**Psychological profile**	**Mean**	**SD**	**Difference of the means for age groups 38–46 and 50-54**	**95% CI for age group difference**	**P for age trend**
**Cesarec-Marke Personality Schedule**					
ACH-achievement	6.21	2.90	0.06	−0.4-0.5	0.48
AFF- affiliation	8.87	2.56	−0.25	−0.7-0.2	**0.04**
AGG-aggression	3.94	2.88	0.32	−0.1-0.8	**0.03**
DST-defence of status	7.35	3.17	−0.11	−0.6-0.4	0.68
GUI – guilt feelings	7.37	3.05	−0.005	−0.5-0.5	0.44
DOM-dominance	6.50	3.29	0.17	−0.4-0.7	0.56
EXH-exhibition	4.58	3.13	0.40	−0.1-0.9	0.15
AUT-autonomy	6.80	2.29	−0.07	−0.4-0.3	0.39
NUR-nurturance	11.89	2.21	−0.58	−0.9 - -0.2	**0.001**
ORD-order	11.43	2.68	−0.20	−0.6-0.2	0.15
SUC-succorance	8.05	2.93	0.29	−0.2-0.8	0.17
Neurotic self-assertiveness	126.52	39.97	0.96	−5.5-7.4	0.89
Dominance	12.31	55.34	4.90	−4.0-13.8	0.21
Aggressive non-conformance	−6.40	26.30	3.38	−0.9-7.6	0.12
Passive dependency	114.92	30.06	0.11	−4.8-5.0	0.87
Sociability	74.30	19.05	−4.74	−7.8 - -1.7	**0.0001**
**Eysenck Personality Inventory**					
Extraversion	11.38	3.31	−0.31	−0.8-0.2	0.54
Neuroticism	8.12	4.60	−0.28	−1.0-0.5	0.74
Lie scale	3.69	1.69	−0.34	−0.6 - -0.1	**0.0004**

Difference <0 implies that the younger group has a lower mean value. P stands for trend tests for linearity over all four age groups (38, 46, 50 and 54 years, respectively).

According to the National Death Registry, 362 (61.5%) of the participants in 1968–69 died between 1968 and 2008. There were no significant linear associations between the 11 personality traits and five personality indices and mortality (Table 3).

**Table 3:** Scale intervals and quartile intervals of personality traits, association with personality trait, highest and lowest quartile, and total death with hazard ratio (HR) and confidence interval (CI) after 40 years of follow-up for women participating in the psychiatric examination of the 1968–69 Population Study of Women in Gothenburg

**Psychological Profile**	**Scale interval**	**Quartile 1**	**Quartile 4**	**HR (CI) Total death**	**HR (CI) Total death quartile 1 vs 2 + 3**	**HR (CI) Total death quartile 4 vs 2 + 3**
**Cesarec-Marke Personality Schedule**						
ACH-achievement	0-14	0-4	9-14	1.07 (0.98-1.17)	0.86 (0.67-1.09)	1.10 (0.85-1.42)
AFF- affiliation	0-15	0-7	11-15	1.05 (0.96-1.15)	0.96 (0.75-1.24)	1.08 (0.84-1.38)
AGG-aggression	0-14	0-2	5-14	1.03 (0.94-1.13)	1.06 (0.82-1.37)	1.11 (0.87-1.42)
DST-defence of status	0-15	0-5	10-15	1.04 (0.95-1.14)	0.85 (0.66-1.09)	0.98 (0.76-1.27)
GUI-guilt feelings	0-15	0-5	10-15	1.08 (0.99-1.18)	0.97 (076–1.26)	1.27 (0.99-1.62)
DOM-dominance	0-15	0-4	9-15	0.94 (0.86-1.02)	1.08 (0.84-1.38)	0.94 (0.73-1.21)
EXH-exhibition	0-14	0-2	7-14	0.98 (0.90-1.08)	1.02 (0.80-1.30)	0.95 (0.73-1.24)
AUT-autonomy	0-18	1-5	9-18	0.97 (0.89-1.06)	0.98 (0.77-1.25)	0.96 (0.74-1.25)
NUR-nurturance	0-15	2-10	14-15	1.03 (0.94-1.13)	0.84 (0.65-1.09)	0.97 (0.76-1.25)
ORD-order	0-15	2-10	14-15	0.99 (0.90-1.09)	1.07 (0.83-1.36)	1.09 (0.85-1.41)
SUC-succorance	0-14	0-6	11-14	0.96 (0.88-1.06)	**1.37 (1.08-1.74)**	1.10 (0.84-1.43)
Neurotic self-assertiveness	0-252	31-97	152-252	1.07 (0.98-1.17)	0.89 (0.68-1.15)	1.06 (0.83-1.35)
Dominance	−118-156	−118- -25	52-156	0.93 (0.85-1.02)	1.14 (0.89-1.46)	0.95 (0.74-1.23)
Aggressive non-conformance	−65-123	−65 - -26	8-123	1.00 (0.90-1.09)	**1.32 (1.03-1.69)**	**1.37 (1.06-1.77)**
Passive dependency	17-181	17-93	137-181	1.00 (0.91-1.10)	1.03 (0.80-1.33)	1.02 (0.79-1.30)
Sociability	−2-120	−2-63	>88	1.05 (0.96-1.15)	0.92 (0.70-1.20)	1.10 (0.86-1.40)
**Eysenck Personality Inventory**						
Extraversion	0-20	2-8	>13	1.06 (0.96-1.16)	0.84 (0.64-1.10)	1.00 (0.79-1.27)
Neuroticism	0-23	0-4	11-23	1.08 (0.98-1.19)	0.83 (0.64-1.09)	1.08 (0.85-1.38)
Lie scale	0-8	0-2	>4	1.01 (0.95-1.08)	1.09 (0.74-1.59)	1.04 (0.78-1.40)

Comparison within separate personality traits between the lowest quartile (quartile 1: Q1) or the highest quartile (quartile 4: Q4) and quartiles 2 + 3 (Q2 + Q3) with age as covariate showed some significant differences: women in the lowest Succorance quartile (Q1) had a significantly higher risk of death (HR = 1.37, CI = 1.08-1.74) than women in Q2 + Q3 (Table 3). Concerning the factor index Aggressive non-conformance, women in the lowest as well as in the highest quartile had significantly higher risks of death (HR = 1.32, CI = 1.03-1.68 and HR = 1.37, CI = 1.96-1.77, respectively) compared to those in Q2 + Q3. Adding marital status, socioeconomic group, education, smoking or hypertension as covariates did not change the results.

**Figure 1:**
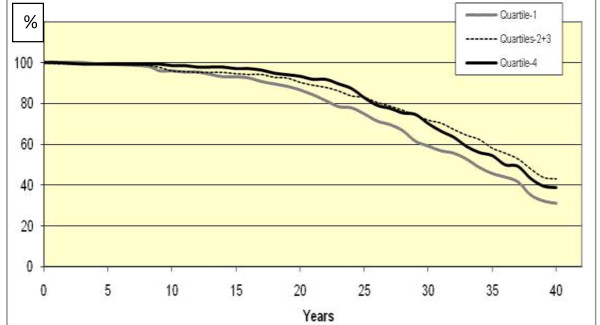
Cumulative survival plot for total mortality for women participating in the psychiatric examination of the1968-69 Population Study of Women in Gothenburg after 40 years of follow-up concerning the personality trait Succorance, quartiles 1, 2 + 3, and 4.

**Figure 2:**
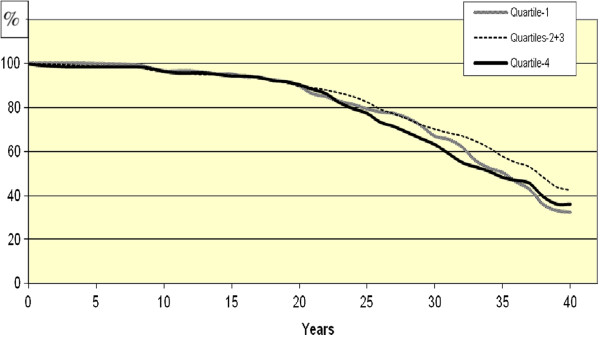
Cumulative survival plot for total mortality for women participating in the psychiatric examination of the 1968–69 Population Study of Women in Gothenburg after 40 years of follow-up concerning the personality trait Aggressive non-conformance, quartiles1, 2 + 3, and 4.

Figures 1 and 2 show survival plots for 40-year survival, comparing Q1, Q2 + Q3 and Q4 for CMPS Succorance and Aggressive non-conformance, in which significant differences were found between the quartiles.

Although no significant reduction of risk over time was found in any of the models, we could observe a tendency of levelling off in risk compared to the reference group during the latest years of follow-up for Q4 of Aggressive non-conformance. On the other hand, Q1 of Aggressive non-conformance showed an opposite pattern: the highest HR was observed in the last 8 years of follow-up.

Interaction between mortality and the effect of personality and age (or birth cohort) was examined for all traits by calculating separate models for younger women (born in 1922 and 1930) and older women (born in 1914 and 1918). Only one factor showed a significant interaction: there was a significantly increased risk of mortality for younger women (born 1930 + 1922) with strong traits associated with Sociability compared to women within the same age group with weaker Sociability traits (HR = 1.59, CI = 1.09-2.32), but for the older age groups there was a conflicting non-significant decrease of mortality risk for women with strong Sociability traits (HR = 0.86, CI = 0.63-1.19; test for interaction p = 0.02).

## Discussion

The main finding of our study was that personality traits did not seem to influence long-term mortality in this population sample of women followed for 40 years from mid- to late life. However, differences in mortality risk could be seen between individuals scoring low or high on two single traits, CMPS Succorance and Aggressive non-conformance, but this did not imply significant influence concerning that single trait on mortality.

A recent Swedish study concerning cohort differences in personality in middle-aged women compared two generations of 38- and 50-year-old women with a lapse of 36 years and showed an increase in the subscales Aggression and Achievement, and, among the factor indices, the greatest increase in Dominance and Aggressive non-conformance [23]. Corresponding changes in the scores between age groups were not confirmed in the present study except for the trend of higher scores concerning Aggression in the younger age groups. However, the largest difference between the age groups in the present study was much smaller – only 12 years.

In the present study the women in the lowest as well as in the highest quartile of the factor index Aggressive non-conformance had a significantly higher risk of death compared to the central quartiles of that index (Q2 + Q3). Aggression and Aggressive non-conformance were considered aspects of hostility, which in turn has emerged as one of the important personality traits for cardiovascular diseases and premature mortality [4,5]. With regard to the secular trends reported in an earlier study [23], further studies of cohorts born later would be of importance. However, in a study of employees in France, mortality was predicted by depressive mood and cognitive hostility in men but not in women [5].

A possible explanation for the weaker or non-existing relation between personality traits and mortality/longevity in women might be that personality in women becomes more circumscribed due to the social constraints exerted by women’s role in society. Taylor et al. suggest that the reason why personality traits do not take full effect on women’s health is the fact that women show less variation in health behaviours [11]. Although women entered the labour market during the second half of the twentieth century when the public sector grew more in Sweden than in other Organisation for European Economic Co-operation countries, they still had the primary responsibility for the family, house and home [25]. The development of personality is influenced by both genetic and environmental factors in a complex adaptive process [2,15,16,26]. As social roles are still quite different for men and women [27], future studies on the association between personality and health outcomes should pay more attention to gender. Simply controlling for sex may obscure this issue [4].

The advantages of the present study were the truly population-based cohort, the high participation rate and the long time span of the follow-up. On the other hand, a limitation of personality studies with a long follow-up time is that the results are limited to the instruments originally used. The NEO Personality Inventory, which has achieved recognition both in theory and practice [13], was not available at the start of the study in 1968. While the CMPS has not been re-evaluated according to the Neo Taxonomy, EPI traits studied here have been shown to be similar to the Five-Factor Model of the constructs neuroticism and extraversion [28].

Only a few studies of the association between personality and mortality have paid attention to the importance of gender in the sense that the results have been stratified according to gender.

In such studies the influence of personality traits on mortality and longevity for women proved to be much weaker than for men or non-existing [5-7,11,29-31]. In the study by Huppert and Whittington, neuroticism had no association with mortality irrespective of gender [6]. One study showed neuroticism to be a significant predictor of mortality concerning men but not women [7]. Friedman discussed how neuroticism may lead people to different pathways [15]. High neuroticism may have depressive effects and lead to non-adherence to healthy behaviour messages, while another possibility is that high neuroticism may lead one to be very vigilant about symptoms needing attention and to comply strictly with medical advice. Perhaps this second pathway is more common among women, as women report more symptoms of disease and psychological distress than men but live longer [15].

The study of cohort changes in women’s personality points to some further important issues concerning research in personality as a predictor of mortality, as the results of personality testing seem to some extent to be context bound [16]. Hence, both the year of personality testing and the age of the persons studied must be taken into account when the results are interpreted. Moreover, results from non-representative populations have to be replicated in population-based studies. A further issue of concern for long-term follow-up studies is the stability of personality traits over the course of a life. Personality traits are considered to be relatively enduring patterns of cognitive, emotional and behavioural factors, but changes have been observed even at midlife and in old age [32,33].

## Conclusions

We could show no linear association between single personality traits and mortality in women followed for 40 years into late life in contrast to what is found in men. One explanation may be that personality in women becomes more circumscribed due to the social constraints exerted by women’s role in society. Our study expands on previous studies with considerably shorter follow-up times.

## Competing interests

The authors declare that they have no competing interests.

## Authors’ contributions

MA, LL, CB, TH and CB designed the study, analysed the results and wrote the paper. VS did the statistical analysis and participated in drafting the paper. MW, EB, IS participated during the analyse and drafting of the paper. All authors read and approved the final manuscript.

## Pre-publication history

The pre-publication history for this paper can be accessed here:

http://www.biomedcentral.com/1472-6874/14/61/prepub
